# Beyond malaria: can intermittent preventive treatment with sulphadoxine-pyrimethamine reduce the number of small vulnerable newborns globally?

**DOI:** 10.1016/S2214-109X(25)00405-X

**Published:** 2025-11-17

**Authors:** Holger W Unger, Ricardo Ataide, Michelle E Roh, Anisur Rahman, Ric N Price, Anna Maria van Eijk, Grant Dorsey, Feiko O ter Kuile, Stephen J Rogerson

**Affiliations:** Global and Tropical Health Division, Menzies School of Health Research, Charles Darwin University, Casuarina, NT, Australia (H W Unger PhD, Prof R N Price MD); Department of Clinical Sciences, Liverpool School of Tropical Medicine, Liverpool, UK (H W Unger, A M van Eijk PhD, Prof F O ter Kuile PhD); Department of Infectious Diseases, University of Melbourne, The Doherty Institute, Melbourne, VIC, Australia (H W Unger, Prof S J Rogerson PhD); Infection and Global Health Division, Walter and Eliza Hall Institute, Parkville, VIC, Australia (R Ataide PhD); Institute for Global Health Sciences, University of California San Francisco, San Francisco, CA, USA (M E Roh PhD); Maternal and Child Health Division, International Centre for Diarrhoeal Disease Research, Dhaka, Bangladesh (A Rahman PhD); Department of Medicine, University of California San Francisco, San Francisco, CA, USA (Prof G Dorsey MD)

## Abstract

Efforts to reduce the global burden of small vulnerable newborns (SVNs) by scaling up existing preventive interventions must be complemented with new preventive approaches to achieve global targets. Intermittent preventive treatment with sulphadoxine-pyrimethamine (IPTp-SP) was originally designed to protect pregnant women from malaria infection, but appears to retain efficacy against low birthweight even when *Plasmodium* infection is absent or the parasite is highly resistant to sulphadoxine-pyrimethamine. This specific effect might occur through the antimicrobial activity of sulphadoxine-pyrimethamine against maternal genitourinary tract infections and pathogenic gut bacteria, direct effects on maternal gut physiology that might reverse environmental enteric dysfunction, or anti-inflammatory actions. Refining our understanding of the pathways underlying the protective efficacy of sulphadoxine-pyrimethamine will require mechanistic studies and placebo-controlled randomised trials of IPTp-SP in non-malarious settings. These studies will be crucial to confirm whether sulphadoxine-pyrimethamine could be considered for reducing the risk of SVNs in settings with high prevalence of SVNs and little or no malaria transmission.

## Introduction

The combination antimicrobial sulphadoxine-pyrimethamine is cheap, widely available, well tolerated, and a key tool in the armamentarium of malaria control. Sulphadoxine-pyrimethamine acts as a synergistic antifolate combination, targeting sequential steps in folate biosynthesis, and is licensed for management of *Pneumocystis* pneumonia, toxoplasmosis, and malaria. The most important application currently is intermittent preventive treatment of malaria in pregnancy (IPTp).^1^ For this purpose, monthly doses of sulphadoxine (1500 mg) and pyrimethamine (75 mg) are administered in pregnancy from the second trimester until birth, to clear existing and prevent new *Plasmodium falciparum* infections. IPTp with sulphadoxine-pyrimethamine (IPTp-SP) is used in 34 malaria-endemic countries in sub-Saharan Africa and in Papua New Guinea.^1,2^ IPTp-SP is one of eight interventions proven to reduce the number of small vulnerable newborns (SVNs), an umbrella term encompassing babies born preterm and/or small for gestational age (SGA), each characterised by different causes, consequences, and thus interventions.^2^ SVNs are at substantial risk of adverse short-term and long-term health outcomes: for example, around 20% of stunting is estimated to originate in utero.^3^

The ability of sulphadoxine-pyrimethamine to limit the deleterious effects of malaria in pregnancy is threatened by the spread of highly resistant *P falciparum* parasites. A large meta-analysis highlighted a persistent, inverse dose–response between the number of sulphadoxine-pyrimethamine doses received and risk of low birthweight (LBW; <2500 g; a widely used surrogate for preterm birth and/or SGA), even in areas with parasites that are highly resistant to sulphadoxine-pyrimethamine, where the antimalarial benefits of sulphadoxine-pyrimethamine are virtually absent.^4^ In smaller-scale observational studies, IPTp-SP was associated with substantial reductions in LBW even in women who were malaria-negative or residing in low malaria transmission settings.^5,6^ These studies suggest sulphadoxine-pyrimethamine has non-malarial effects on pregnancy outcomes.

Many SVNs are born in southern Asia (eg, India, Pakistan, Bangladesh, and Nepal), where malaria transmission is low or absent and IPTp-SP is currently not recommended.^7^ Current strategies to reduce SVN rates in these settings focus on nutritional education and supplementation with energy, protein, iron, and folic acid during pregnancy.^2^ Although these strategies have led to reductions in SVN rates, and their implementation should therefore be accelerated, additional tools are needed to further the reduction in SVN rates. We hypothesise that using IPTp-SP to reduce the number of SVNs in populations with high rates, or in individuals with risk factors that the non-malarial effects of sulphadoxine-pyrimethamine can address, could close this gap and accelerate reduction of SVN rates globally. We advocate for a framework shift, whereby the research community endeavours to quantify and characterise the non-malarial effects of IPTp-SP to establish if it could be repurposed to reduce multicausal SVNs rather than exclusively serve as a malaria prevention strategy.

In this Viewpoint, we discuss the current evidence to explore three related questions. First, what is the epidemiological evidence that IPTp-SP reduces SVNs through non-malarial effects? Second, what are the mechanisms by which sulphadoxine-pyrimethamine could operate to reduce SVNs? And third, what research is required to establish whether sulphadoxine-pyrimethamine use could be expanded to reduce the number of SVNs in high-prevalence settings with low or no malaria transmission?

## Epidemiological evidence that IPTp-SP reduces SVNs through non-malarial effects

Signals of non-malarial effects of sulphadoxine-pyrimethamine emerged from observational and clinical trial data in two contexts: sulphadoxine-pyrimethamine appears to prevent LBW in observational studies from areas with low-to-no malaria transmission, and in women who tested negative for malaria infection throughout pregnancy; and randomised trials suggest sulphadoxine-pyrimethamine appears to have better protective efficacy against LBW in malaria-endemic areas with widespread high-grade sulphadoxine-pyrimethamine resistance than IPTp regimens with superior antimalarial efficacy.

An individual participant data meta-analysis of approximately 14 000 pregnancies derived from 13 cohort studies in eight countries with moderate-to-high malaria transmission highlighted possible non-malarial effects of sulphadoxine-pyrimethamine leading to less LBW (table).^9^ Specifically, analyses suggested that if all women received three or more doses of IPTp-SP, the incidence of LBW could be reduced from 9·9% to 6·9%, a 30% reduction in relative risk. The absolute risk of LBW could be reduced by 3%, which aligns with the WHO 2025 Global Nutrition Target for reducing LBW, and is far greater than the estimated impact of complete elimination of malaria infection (<1% reduction in absolute risk).^14^ Non-malarial effects on SVNs were reported in a prospective cohort study from Zambia, in which pregnant women who took at least three doses of IPTp-SP had 57% lower odds of an adverse pregnancy outcome (ie, composite of stillbirth, LBW, preterm birth, and SGA) compared with those who took one dose or less; this effect appeared to be partly driven by sulphadoxine-pyrimethamine preventing preterm birth, and appeared to be independent of malaria infection.^5^ In a low malaria transmission setting in Papua New Guinea, babies of women who received at least three doses of IPTp-SP had a 61% reduction in the odds of LBW and a 151 g higher mean birthweight compared babies of women who received one dose or less, and fewer preterm births were reported.^6^ However, these observational studies are prone to inherent biases that could affect the validity of their findings. Few women had early ultrasound dating—crucial to discriminate preterm birth from SGA—and the number of sulphadoxine-pyrimethamine doses correlated highly with the number of antenatal visits and gestational age at first sulphadoxine-pyrimethamine administration.^6^ We suspect that women receiving more sulphadoxine-pyrimethamine doses could systematically differ from those receiving fewer doses, both in their underlying characteristics and by receiving more antenatal care. Additionally, preterm delivery reduces opportunity for multiple doses of sulphadoxine-pyrimethamine due to earlier delivery, creating reverse causation bias, and immortal time bias is often not adjusted for in observational research.

Effect estimates of the non-malarial impacts of sulphadoxine-pyrimethamine derived from clinical trials are probably more robust, as randomisation should balance the number of antenatal visits and timing of dosing between trial groups. Summary estimates from a meta-analysis of seven clinical trials showed that three or more doses of sulphadoxine-pyrimethamine was associated with a higher mean birthweight and less LBW than two doses; this was observed in areas with low and high resistance to sulphadoxine-pyrimethamine.^8^ Mediation analysis of individual participant data from six trials comparing IPTp-SP and IPTp with dihydroartemisinin-piperaquine (IPTp-DP) indicated substantial non-malarial effects of IPTp-SP on birthweight (table).^12^ Although dihydroartemisinin-piperaquine was superior to sulphadoxine-pyrimethamine in preventing malaria (50–80% reduction in malaria incidence), mothers who received IPTp-SP still had bigger babies. In this analysis, the non-malarial effect of sulphadoxine-pyrimethamine on birthweight (0·15 increase in birthweight z-scores) was substantially greater than the 0·01 increase associated with the antimalarial effect of dihydroartemisinin-piperaquine, and mean gestational age did not differ.^12^ In a trial that used serial fetal biometry to date pregnancies, z-scores for estimated fetal weight were significantly higher and the relative risks of LBW and SGA were on average ~30% and ~23% lower, respectively, in women randomly assigned to receive sulphadoxine-pyrimethamine compared with those who received dihydroartemisinin-piperaquine or dihydroartemisinin-piperaquine plus azithromycin; preterm birth rates did not differ between groups receiving sulphadoxine-pyrimethamine and dihydroartemisinin-piperaquine.^11^ Although these findings provide compelling evidence to support the non-malarial effects of sulphadoxine-pyrimethamine on SGA, several trials used active comparators, so observed differences in outcomes (or lack thereof) could partly reflect opposing effects of the regimens, including potential negative effects of the comparator (based on evidence from a preprint paper).^15^

## Possible non-malarial mechanisms by which sulphadoxine-pyrimethamine might reduce SVNs

We postulate that sulphadoxine-pyrimethamine reduces SVNs through three distinct non-malarial mechanisms: effects on maternal gut physiology and microbiome, activity against pathogenic infections other than malaria, and anti-inflammatory actions (figure).

### Effects on gestational weight gain

In several clinical trials, IPTp-SP and sulphadoxine-pyrimethamine-based IPTp substantially improved gestational weight gain, a predictor of birthweight.^11,13–10^ In Papua New Guinea, women who received IPTp with sulphadoxine-pyrimethamine plus azithromycin (~3 doses) had a 60 g/week higher gestational weight gain than women who received a single treatment course of sulphadoxine-pyrimethamine plus chloroquine at the enrolment visit.^13^ This improved gestational weight gain partially mediated the higher mean birthweights of babies of women receiving sulphadoxine-pyrimethamine plus azithromycin, and undernourished women benefitted most.^13^ However, women in the control group also received one dose of sulphadoxine-pyrimethamine, thus, co-administered azithromycin in the intervention group could have exerted independent effects on gestational weight gain.^10^ Meta-analysis of six trials comparing IPTp-SP with IPTp-DP indicated that the superior effect of sulphadoxine-pyrimethamine on SGA was partially mediated by a greater effect on gestational weight gain.^12^ IPTp-SP also appeared to improve mid-upper-arm circumference and body mass index in the third trimester, at birth, or 6–8 weeks postpartum.^11,13,16^ In Malawi, monthly sulphadoxine-pyrimethamine resulted in approximately 78 g/week higher weight gain compared with two doses in HIV-positive women.^10^ A recent meta-analysis found total gestational weight gain was approximately 700 g higher amongst recipients of IPTp-SP than with chloroquine prophylaxis.^17^

### Effects on gastrointestinal infection and health

Molecular screening for bacterial and protozoal enteric pathogens nested into a trial comparing IPTp-SP with IPTp-DP indicated that improvements in gestational weight gain and birthweight could in part be achieved by reducing women’s risk of acquiring *Escherichia coli* pathotypes implicated in acute and chronic diarrhoeal illness;^16^ this benefit was restricted to women who were uninfected with *E coli* pathotypes before starting IPTp.^16^ No studies we found to date have evaluated the effects of sulphadoxine-pyrimethamine on the maternal gut microbiome, an important gap as it predicts both gestational weight gain and birthweight,^18^ and might explain the longer-term benefits of IPTp-SP for child health, including less postnatal growth faltering.^12,13,19,20^ A substantial proportion of organisms colonising the infant gut derive from the maternal gut microbiome,^21^ and maternal gut microbiome composition also predicts weight for age at 1 month of life.^18^ Alterations of the maternal gut microbiome induced by sulphadoxine-pyrimethamine could therefore alter the trajectory of community assembly in infants, which could in turn improve infant growth.

Sulphadoxine-pyrimethamine might also have direct therapeutic effect on gut health. In an intestine-on-a-chip model, perfusion with an estimated duodenal dose equivalent to one tablet of sulphadoxine-pyrimethamine (500 mg/25 mg) reversed multiple absorptive duodenal abnormalities associated with enteric dysfunction, including villus blunting, reduced mucous production, impaired nutrient absorption, and inflammatory cytokine production.^22^ Although the burden of environmental enteric dysfunction in pregnancy in under-resourced settings is poorly described, many women are probably at risk due to food insecurity, poor diet, and inadequate sanitation.^23^ Reversing environmental enteric dystrophy—thus improving nutrient absorption by the small intestine and supporting the caloric demands of pregnancy—could be a crucial mechanism by which sulphadoxine-pyrimethamine improves fetal growth. Whether sulphadoxine-pyrimethamine prevents or reverses intestinal permeability (leaky gut), a cause of systemic inflammation due to the translocation of pathogen-derived proteins from the gut into the blood stream, is unknown.

### Effects on genitourinary and respiratory tract infections

Maternal *Chlamydia trachomatis* infection has been associated with preterm birth, SGA, and LBW.^24,25^ In malaria-negative women infected with chlamydia, frequent dosing of sulphadoxine-pyrimethamine halved the risk of LBW compared with uninfected women,^5^ suggesting that sulphadoxine-pyrimethamine affects this sexually transmitted infection (STI). In a trial where all women received IPTp-SP, *C trachomatis* prevalence fell from 21·8% to 9·6% after 1 month of sulphadoxine-pyrimethamine in the syndromic STI management group, compared with 24·1% to 4·1% in the test-and-treat group (12·2% *vs* 80·2% received targeted STI treatment, respectively).^26^ Similarly, when women received sulphadoxine-pyrimethamine plus chloroquine (not known to be effective against chlamydia) or sulphadoxine-pyrimethamine plus azithromycin, the prevalence of chlamydia fell from 11% at baseline to 4% at the subsequent visit across both trial groups.^27^ In another trial comparing sulphadoxine-pyrimethamine with dihydroartemisinin-piperaquine alone or combined with a single course of azithromycin at enrolment, chlamydia prevalence at antenatal booking was 14%, and only 2% of participants who received sulphadoxine-pyrimethamine were infected at delivery, compared with 10% and 7% in dihydroartemisinin-piperaquine and dihydroartemisinin-piperaquine plus azithromycin arms, respectively.^11^ The observed effect of sulphadoxine-pyrimethamine could be through preventing reinfection by repeated dosing. These findings mirror research indicating that sulfonamides retain activity against *C trachomatis*.^28^ In Uganda, IPTp-SP was found to reduce not only chlamydia but also group B *Streptocococcus* carriage and respiratory non-malarial febrile illness in pregnant women compared with women treated with IPTp-DP,^15,29^ consistent with its established activity against some respiratory pathogens.^30^ In the same trial, however, sulphadoxine-pyrimethamine did not decrease clinically suspected febrile urinary tract infections; asymptomatic infections were not investigated.

### Immunomodulatory effects

Elevated levels of the acute phase protein alpha-1-acid glycoprotein (AGP) in maternal plasma at first antenatal visit or at birth are associated with LBW, preterm birth, SGA, fetal loss,^23,31,32^ and poor gestational weight gain.^31^ In Malawi and Papua New Guinea, elevated AGP at birth doubled the risk of adverse birth outcomes, especially for preterm birth, but only in women not receiving IPTp-SP.^31,33^ IPTp-SP also appeared to abrogate the association between raised concentration of the anti-angiogenic factor soluble fms-like tyrosine kinase-1, associated with pre-eclampsia, and preterm birth.^33^ Protection against infection and inflammation through frequent sulphadoxine-pyrimethamine dosing could further explain the associations between maternal iron status and birthweight. Although a doubling of ferritin levels, a marker of iron stores measured at antenatal enrolment, was associated with a 20% reduction in LBW risk in recipients of IPTp-SP, the same ferritin difference in recipients of a single dose of sulphadoxine-pyrimethamine plus chloroquine was associated with a 15% increase in LBW,^34^ and this effect was independent of anaemia. In a recent meta-analysis, dihydroartemisinin-piperaquine was more effective than sulphadoxine-pyrimethamine against moderate maternal anaemia,^12^ suggesting that anaemia reduction is not an important mechanism underlying the effects of sulphadoxine-pyrimethamine on maternal weight gain or fetal growth.

## Research required to establish whether sulphadoxine-pyrimethamine use could be expanded to reduce SVNs in high-burden settings with low or no malaria transmission

The most definitive approach to quantifying and exploring mechanisms underpinning putative non-malarial effects on maternal, birth and long-term infant outcomes would require randomised trials of IPTp-SP versus placebo in non-malarious areas with a high burden of SVNs. Given the preliminary data on effects of sulphadoxine-pyrimethamine on maternal anthropometric outcomes and gut function, settings where maternal undernutrition is a principal driver of SVNs are of particular interest. These trials, which should use the SVN framework to facilitate a more refined understanding of the effects of sulphadoxine-pyrimethamine on SVN subtypes and related risk factors,^2^ would permit targeted assessment of non-malarial effects by eliminating confounding from differential antenatal clinic utilisation and potential competing non-malarial effects of comparator anti-malarials such as dihydroartemisinin-piperaquine.^35,36^ Studies should characterise further the non-malarial mechanisms of action of sulphadoxine-pyrimethamine and optimise sulphadoxine-pyrimethamine dosing and frequency. To increase understanding of treatment effects on maternal gut health and gestational weight gain, and through these on birth outcomes, ancillary studies should measure: maternal stool and plasma biomarkers of enteropathy (eg, faecal myeloperoxidase, neopterin, and alpha-1-antitrypsin); gut leak and inflammation; and treatment-associated effects on the gut microbiome across trial groups.^22^ This work should be complemented with intestine-on-a-chip studies that simulate effect of repeated treatments with full-dose sulphadoxine-pyrimethamine (1500/75 mg) on gut health and establish longevity of the restorative effect. Treatment-associated effects on bacteriuria, STIs, and the vaginal microbiome also warrant further investigation.^37^ For example, effects of sulphadoxine-pyrimethamine on *Lactobacillus* spp, such as *L crispatus*, which confer an optimal vaginal microbial state and reduced risk of preterm birth, need to be explored.^38^ Vaginal dysbiosis and STIs are associated with inflammation, and sulphadoxine-pyrimethamine-associated effects on the vaginal microbiome or placental inflammation might mediate reductions in SGA and preterm birth. Concurrent modelling studies will crucially inform where and for whom IPTp-SP could achieve substantial reductions in the number of SVNs, drawing on validated estimates of: sulphadoxine-pyrimethamine-associated reductions in the risk of an SVN subtype attributable to a particular risk factor; the prevalence of sulphadoxine-pyrimethamine-modifiable risk factors in the population; and the incidence of adverse birth outcomes to inform implementation strategies. These enquiries should be complemented with assessments of cost-effectiveness, acceptability, infant growth and anthropometry, and effects of expanding IPTp-SP to non-malarious areas, depending on local antimicrobial resistance patterns.^39^

## Conclusion

The key to reducing the global burden of SVNs is affordable, acceptable, and easily administered interventions that can be implemented quickly. If IPTp-SP reduces SVNs through non-malarial effects, dissecting its effects on SVN subtypes and the underlying mechanisms could enable more targeted use, as sulphadoxine-pyrimethamine is unlikely to affect all subtypes equally. The effects of IPTp-SP in low and non-malaria endemic settings with a high burden of SVNs should therefore be investigated as a matter of priority. Elucidating the non-malarial effects of sulphadoxine-pyrimethamine on reducing SVNs could be a pivotal step towards healthier mothers, babies and children globally.

## Figures and Tables

**Figure:**
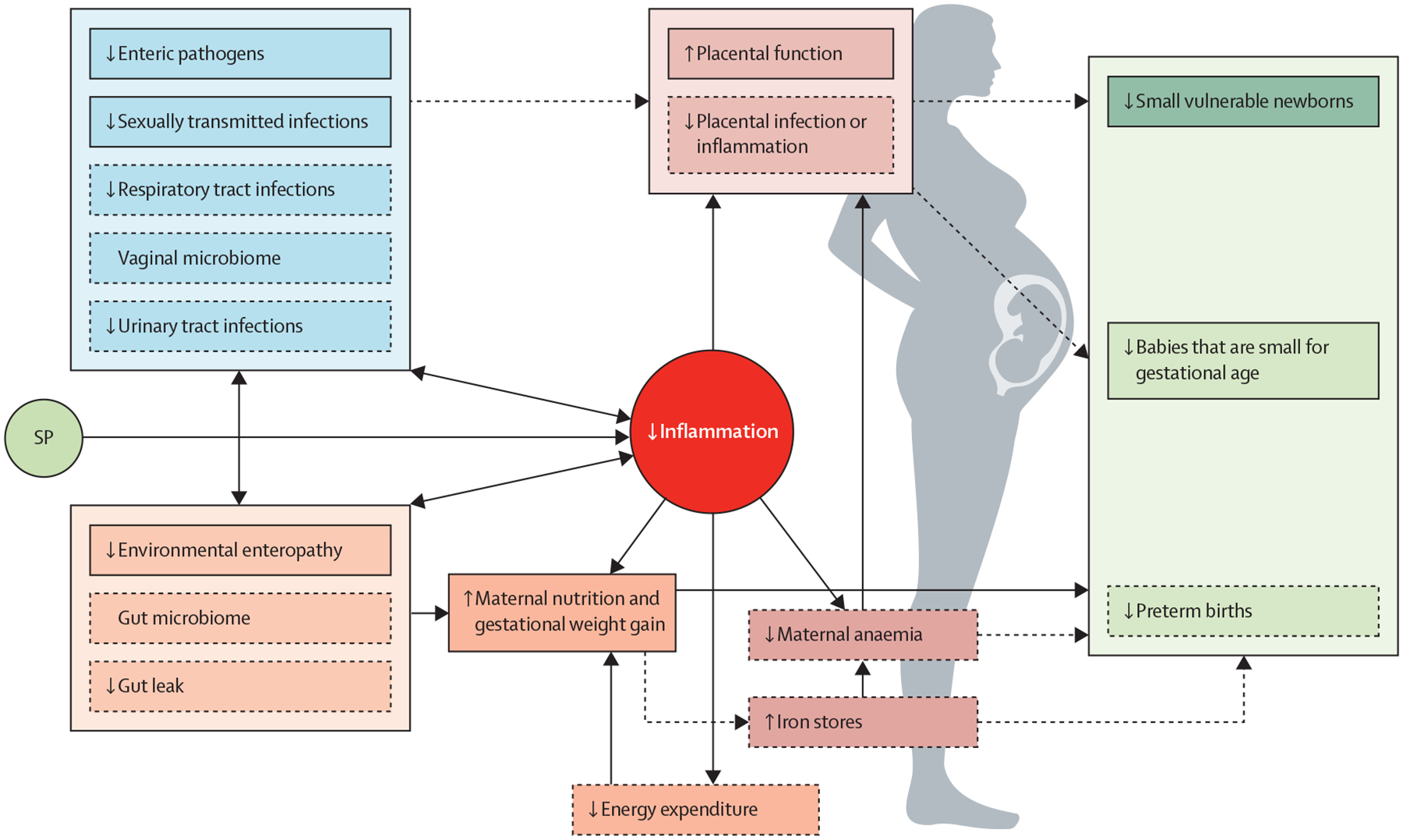
Emerging and speculative non-malarial mechanisms by which intermittent preventive treatment in pregnancy with sulphadoxine-pyrimethamine mediates improvements in birth outcomes Components with scant evidence are marked with dashed borders. SP=sulphadoxine-pyrimethamine.

**Table:** Non-malarial effects of sulphadoxine-pyrimethamine on maternal and birth outcomes

	Location	Year of study	Study design	Transmission rate	Number of participants	Mean birth weight	LBW	Preterm birth	SGA	Anaemia	GWG	Other findings
Kayentao, 2013^8^	Kenya, Tanzania, Malawi, Zambia, Burkina Faso, and Mali	1994–2008	Aggregate data meta-analysis of seven trials	Low to high	6281	Increased	Decreased	No change	..	Decreased	..	Meta-analysis of clinical trial showing that ≥3 doses of SP increased birthweight and reduced LBW relative to 2-dose regimens and observed in areas with low and high resistance to SP
Cates, 2018^9^	Multiple countries*	1996–2015	Individual participant data meta-analysis	Moderate to high	14 633	..	Decreased	..	..	..	..	Effect of SP on LBW outweighs effect of malaria on LBW three-fold
Cellich, 2024^6^	Papua New Guinea	2022	Cohort study (retrospective)	Low	1140	Increased	Decreased	Decreased	..	Decreased	..	Effects observed in low-transmission to no-transmission setting
Chico, 2017^5^	Zambia	2013–14	Cohort study (prospective)	Moderate to high	1086†	..	Decreased	Decreased	No change	..	..	Effects observed in absence of malarial infection
Luntamo, 2025^10^	Malawi	2003–06	RCT: IPTp-SP (two doses) vs IPTp-SP (three doses) *vs* IPT-SP (three doses) plus azithromycin	Moderate to high	1320	No change	Decreased	No change	..	No change	Increased	Reduction in LBW, neonatal growth faltering and stunting with three or more doses of SP *vs* two doses; effects on GWG most pronounced in women who were HIV-positive
Madanitsa, 2023^11^	Kenya, Uganda, and Malawi	2018–19	RCT: IPTp-DP (azithromycin) vsIPTp-SP	Moderate to high	4680	Increased	Decreased	No change	Decreased	No change	Increased	High-grade SP resistance; IPTp-DP provided superior antimalarial protection
Roh, 2025^12^	Kenya, Malawi, Tanzania, and Uganda	2012–19	Individual participant data meta-analysis: IPTp-DP vs IPTp-SP	Moderate to high	6646	Increased	No change	No change	Decreased	Decreased	Increased	Superior effect of SP on SGA was primarily observed in multigravidae; maternal MUAC, GWG, and early infant growth outcomes were greater in IPTp-SP groups
Unger, 2016^13^	Papua New Guinea	2009–13	RCT: SP-CQ‡ *vs* IPTp-SP plus azithromycin	Low to moderate	2793	Increased	Decreased	Decreased	..	No change	Increased	Maternal postnatal BMI and MUAC were greater in IPTp-SP plus azithromycin group
van Eijk, 2025^4^	Multiple countries§	1999–2021	Individual participant and aggregate data meta-analysis	Moderate to high	149 941	..	Decreased	Decreased	..	..	..	Three doses of IPTp-SP reduced LBW by 25–45% *vs* two doses, despite high-grade resistance

Clinical studies that indicate that non-malarial effects of intermittent preventive treatment with sulphadoxine-pyrimethamine mediate improvements in maternal health and birth outcomes. CQ=chloroquine. GWG=gestational weight gain. IPTp=intermittent preventive treatment of malaria in pregnancy. LBW=low birthweight. MUAC=mid-upper-arm circumference. SGA=small for gestational age. SP=sulphadoxine-pyrimethamine. RCT=randomised controlled trial.

* Benin, Burkina Faso, Democratic Republic of the Congo, Ghana, Kenya, Malawi, Papua New Guinea, and Tanzania.

† Approximately 15% of participants were HIV-positive.

‡ Single treatment course of SP plus chloroquine at first antenatal visit.

§ 68 studies from central and west Africa and 54 from east and southern Africa.
